# (*E*)-*N*′-(3,5-Dibromo-2-hydroxy­benzyl­idene)-4-hydroxy­benzohydrazide monohydrate

**DOI:** 10.1107/S1600536808030304

**Published:** 2008-09-27

**Authors:** Xiao-Ya Wang, Guo-Biao Cao, Tao Yang

**Affiliations:** a Department of Biology, Ankang University, Ankang Shanxi 725000, People’s Republic of China; b Department of Chemistry, Ankang University, Ankang Shanxi 725000, People’s Republic of China

## Abstract

The title compound, C_14_H_10_Br_2_N_2_O_3_·H_2_O, was synthesized by the reaction of 3,5-dibromo-2-hydroxy­benzaldehyde with an equimolar amount of 4-hydroxy­benzohydrazide in methanol. The structure comprises a Schiff base unit and a water mol­ecule of crystallization. The dihedral angle between the benzene rings in the Schiff base is 1.3 (3)°. In the crystal structure, mol­ecules are linked through inter­molecular O—H⋯O and N—H⋯O hydrogen bonds, with the water mol­ecule serving as both donor and acceptor. As a result, layers are formed, which are approximately parallel to the *bc* plane.

## Related literature

For related structures, see: Cao (2007*a* ▶,*b* ▶); Yang *et al.* (2008 ▶).
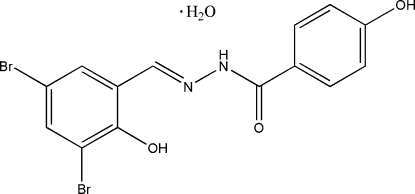


## Experimental

### 

#### Crystal data

C_14_H_10_Br_2_N_2_O_3_·H_2_O
*M* *_r_* = 432.08
Monoclinic, 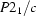

*a* = 6.9840 (16) Å
*b* = 12.678 (3) Å
*c* = 17.722 (4) Å
β = 96.999 (4)°
*V* = 1557.4 (6) Å^3^
*Z* = 4
Mo *K*α radiation
μ = 5.22 mm^−1^
*T* = 298 (2) K
0.23 × 0.23 × 0.22 mm

#### Data collection

Bruker SMART CCD area-detector diffractometer
Absorption correction: multi-scan (*SADABS*; Bruker, 2001 ▶) *T*_min_ = 0.307, *T*_max_ = 0.318
12695 measured reflections
3366 independent reflections
2045 reflections with *I* > 2σ(*I*)
*R*_int_ = 0.075

#### Refinement

*R*[*F* ^2^ > 2σ(*F* ^2^)] = 0.046
*wR*(*F* ^2^) = 0.106
*S* = 0.99
3366 reflections
210 parameters
4 restraints
H atoms treated by a mixture of independent and constrained refinement
Δρ_max_ = 0.42 e Å^−3^
Δρ_min_ = −0.42 e Å^−3^

### 

Data collection: *SMART* (Bruker, 2007 ▶); cell refinement: *SAINT* (Bruker, 2007 ▶); data reduction: *SAINT*; program(s) used to solve structure: *SHELXTL* (Sheldrick, 2008 ▶); program(s) used to refine structure: *SHELXTL* molecular graphics: *SHELXTL*; software used to prepare material for publication: *SHELXTL*.

## Supplementary Material

Crystal structure: contains datablocks global, I. DOI: 10.1107/S1600536808030304/bh2193sup1.cif

Structure factors: contains datablocks I. DOI: 10.1107/S1600536808030304/bh2193Isup2.hkl

Additional supplementary materials:  crystallographic information; 3D view; checkCIF report

## Figures and Tables

**Table 1:** Hydrogen-bond geometry (Å, °)

*D*—H⋯*A*	*D*—H	H⋯*A*	*D*⋯*A*	*D*—H⋯*A*
O1—H1⋯N1	0.82	1.86	2.578 (4)	146
O3—H3⋯O2^i^	0.82	1.83	2.642 (4)	173
O4—H4*A*⋯O3^ii^	0.847 (10)	2.038 (14)	2.878 (4)	171 (5)
O4—H4*B*⋯O1^i^	0.851 (10)	2.24 (3)	2.969 (5)	144 (4)
N2—H2⋯O4^iii^	0.898 (10)	2.01 (2)	2.874 (5)	162 (5)

Symmetry codes: (i) 
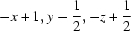
; (ii) 
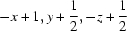
; (iii) 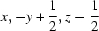.

**Fig. 1.**
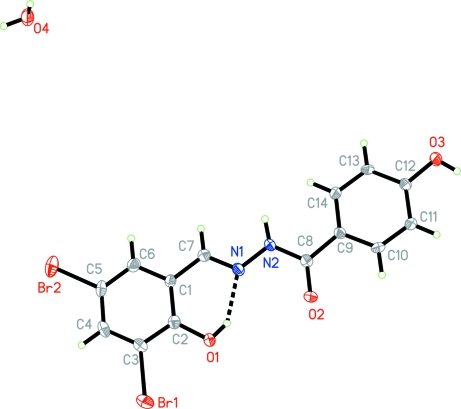
The molecular structure of (I) with ellipsoids drawn at the 30% probability level. Water and main molecule are placed in two different asymmetric units.

**Fig. 2.**
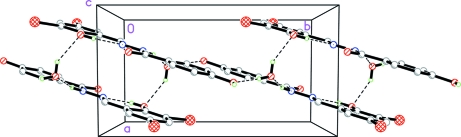
The molecular packing of (I), viewed along the c axis. Hydrogen bonds are drawn as dashed lines.

## supplementary crystallographic information

### Comment

We have recently reported some transition metal complexes with Schiff base ligands (Cao, 2007*a*; Cao, 2007*b*). We report herein the crystal structure of the title compound, (I), derived from the reaction of 3,5-dibromo-2-hydroxybenzaldehyde with an equimolar quantity of 4-hydroxybenzohydrazide in methanol.

The compound (I), Fig. 1, comprises a Schiff base unit and a water molecule of crystallization. The dihedral angle between the two benzene rings in the Schiff base unit is 1.3 (3)°. All bond lengths are comparable to the similar compound, 3-bromo-*N*'-[(*E*)-4-hydroxybenzylidene]benzohydrazide, which we reported previously (Yang *et al.*, 2008). In the crystal structure, molecules are linked through intermolecular hydrogen bonds of types O—H···O and N—H···O (Table 1), forming 2D layers approximately parallel to the *bc* plane, as shown in Fig. 2.

### Experimental

The compound was prepared by refluxing equimolar quantities of 3,5-dibromo-2-hydroxybenzaldehyde with 4-hydroxybenzohydrazide in methanol. Colorless block crystals were formed when the solution was evaporated in air over five days.

### Refinement

Water H atoms and H2 were located in a difference map and refined isotropically, with O—H, N—H, and H···H distances restrained to 0.85 (1), 0.90 (1), and 1.37 (2) Å, respectively. The other H atoms were placed in idealized positions and constrained to ride on their parent atoms, with C—H distances of 0.93 Å and O—H distance of 0.82 Å, and with *U*_iso_(H) set at 1.2*U*_eq_(C) and 1.5*U*_eq_(O).

### Crystal data

**Table:**

C_14_H_10_Br_2_N_2_O_3_·H_2_O	*F*(000) = 848
*M**_r_* = 432.08	*D*_x_ = 1.843 Mg m^−^^3^
Monoclinic, *P*2_1_/*c*	Mo *K*α radiation, λ = 0.71073 Å
Hall symbol: -P 2ybc	Cell parameters from 1545 reflections
*a* = 6.9840 (16) Å	θ = 2.3–24.9°
*b* = 12.678 (3) Å	µ = 5.22 mm^−^^1^
*c* = 17.722 (4) Å	*T* = 298 K
β = 96.999 (4)°	Block, colourless
*V* = 1557.4 (6) Å^3^	0.23 × 0.23 × 0.22 mm
*Z* = 4	

### Data collection

**Table:**

Bruker SMART CCD area-detector diffractometer	3366 independent reflections
Radiation source: fine-focus sealed tube	2045 reflections with *I* > 2σ(*I*)
graphite	*R*_int_ = 0.075
ω scans	θ_max_ = 27.0°, θ_min_ = 2.0°
Absorption correction: multi-scan (SADABS; Bruker, 2001)	*h* = −8→8
*T*_min_ = 0.308, *T*_max_ = 0.318	*k* = −16→15
12695 measured reflections	*l* = −22→22

### Refinement

**Table:**

Refinement on *F*^2^	Primary atom site location: structure-invariant direct methods
Least-squares matrix: full	Secondary atom site location: difference Fourier map
*R*[*F*^2^ > 2σ(*F*^2^)] = 0.046	Hydrogen site location: inferred from neighbouring sites
*wR*(*F*^2^) = 0.106	H atoms treated by a mixture of independent and constrained refinement
*S* = 1.00	*w* = 1/[σ^2^(*F*_o_^2^) + (0.0361*P*)^2^] where *P* = (*F*_o_^2^ + 2*F*_c_^2^)/3
3366 reflections	(Δ/σ)_max_ = 0.001
210 parameters	Δρ_max_ = 0.42 e Å^−^^3^
4 restraints	Δρ_min_ = −0.42 e Å^−^^3^
0 constraints	

### Fractional atomic coordinates and isotropic or equivalent isotropic displacement parameters (Å^2^)

**Table:**

	*x*	*y*	*z*	*U*_iso_*/*U*_eq_	
Br1	0.07183 (8)	0.86697 (4)	−0.00493 (3)	0.0601 (2)	
Br2	0.05286 (8)	0.71867 (5)	−0.30593 (3)	0.0664 (2)	
O1	0.1871 (5)	0.6446 (2)	0.03203 (16)	0.0405 (7)	
H1	0.2190	0.5834	0.0420	0.061*	
O2	0.3386 (4)	0.4075 (2)	0.14777 (16)	0.0426 (8)	
O3	0.5946 (6)	−0.0702 (2)	0.20309 (16)	0.0594 (10)	
H3	0.6086	−0.0733	0.2497	0.089*	
O4	0.4571 (5)	0.2539 (3)	0.39995 (18)	0.0494 (8)	
N1	0.2698 (5)	0.4520 (3)	0.00247 (19)	0.0323 (8)	
N2	0.3257 (5)	0.3529 (3)	0.02714 (19)	0.0336 (8)	
C1	0.1784 (6)	0.5746 (3)	−0.0955 (2)	0.0301 (10)	
C2	0.1564 (6)	0.6572 (3)	−0.0440 (2)	0.0336 (10)	
C3	0.1013 (6)	0.7560 (3)	−0.0737 (3)	0.0388 (11)	
C4	0.0691 (6)	0.7749 (4)	−0.1503 (3)	0.0422 (11)	
H4	0.0323	0.8414	−0.1687	0.051*	
C5	0.0927 (6)	0.6928 (4)	−0.1997 (2)	0.0385 (11)	
C6	0.1470 (6)	0.5942 (4)	−0.1731 (2)	0.0398 (11)	
H6	0.1628	0.5401	−0.2073	0.048*	
C7	0.2368 (6)	0.4704 (3)	−0.0687 (2)	0.0358 (10)	
H7	0.2504	0.4166	−0.1033	0.043*	
C8	0.3580 (6)	0.3355 (3)	0.1033 (2)	0.0311 (10)	
C9	0.4152 (6)	0.2264 (3)	0.1270 (2)	0.0292 (9)	
C10	0.4475 (7)	0.2041 (3)	0.2037 (2)	0.0430 (12)	
H10	0.4294	0.2573	0.2383	0.052*	
C11	0.5056 (7)	0.1059 (3)	0.2311 (2)	0.0438 (12)	
H11	0.5262	0.0933	0.2832	0.053*	
C12	0.5325 (7)	0.0275 (3)	0.1807 (2)	0.0385 (11)	
C13	0.4980 (7)	0.0477 (3)	0.1040 (2)	0.0528 (14)	
H13	0.5146	−0.0059	0.0696	0.063*	
C14	0.4397 (7)	0.1452 (3)	0.0773 (2)	0.0462 (12)	
H14	0.4163	0.1569	0.0252	0.055*	
H2	0.356 (7)	0.307 (3)	−0.008 (2)	0.080*	
H4A	0.436 (6)	0.309 (3)	0.373 (3)	0.080*	
H4B	0.5787 (19)	0.245 (4)	0.408 (3)	0.080*	

### Atomic displacement parameters (Å^2^)

**Table:**

	*U*^11^	*U*^22^	*U*^33^	*U*^12^	*U*^13^	*U*^23^
Br1	0.0799 (4)	0.0359 (3)	0.0643 (4)	0.0158 (3)	0.0076 (3)	−0.0051 (3)
Br2	0.0707 (4)	0.0883 (5)	0.0380 (3)	0.0029 (3)	−0.0022 (2)	0.0233 (3)
O1	0.061 (2)	0.0292 (17)	0.0304 (16)	0.0048 (16)	0.0005 (15)	0.0034 (13)
O2	0.066 (2)	0.0267 (16)	0.0348 (17)	0.0075 (15)	0.0035 (15)	−0.0053 (14)
O3	0.122 (3)	0.0215 (17)	0.0333 (18)	0.0134 (19)	0.003 (2)	0.0046 (14)
O4	0.070 (2)	0.041 (2)	0.0359 (18)	−0.0058 (18)	0.0031 (17)	0.0069 (15)
N1	0.035 (2)	0.026 (2)	0.034 (2)	0.0008 (16)	0.0007 (16)	0.0021 (16)
N2	0.046 (2)	0.023 (2)	0.031 (2)	0.0049 (17)	0.0024 (17)	0.0037 (15)
C1	0.032 (2)	0.032 (2)	0.026 (2)	0.0011 (19)	0.0028 (18)	0.0022 (19)
C2	0.032 (2)	0.035 (3)	0.033 (2)	0.001 (2)	0.0032 (19)	0.004 (2)
C3	0.039 (3)	0.030 (2)	0.047 (3)	0.004 (2)	0.004 (2)	0.001 (2)
C4	0.041 (3)	0.035 (3)	0.050 (3)	0.006 (2)	0.001 (2)	0.016 (2)
C5	0.035 (2)	0.049 (3)	0.030 (2)	0.000 (2)	0.0011 (19)	0.015 (2)
C6	0.042 (3)	0.045 (3)	0.032 (2)	0.004 (2)	0.001 (2)	0.000 (2)
C7	0.043 (3)	0.032 (3)	0.032 (2)	0.003 (2)	0.002 (2)	−0.0007 (19)
C8	0.029 (2)	0.029 (2)	0.034 (2)	−0.0019 (19)	0.0004 (19)	0.000 (2)
C9	0.034 (2)	0.023 (2)	0.030 (2)	−0.0003 (19)	0.0010 (18)	0.0003 (18)
C10	0.067 (3)	0.033 (3)	0.030 (2)	0.007 (2)	0.006 (2)	−0.010 (2)
C11	0.073 (3)	0.032 (3)	0.026 (2)	0.009 (2)	0.006 (2)	0.001 (2)
C12	0.064 (3)	0.019 (2)	0.033 (3)	0.003 (2)	0.009 (2)	0.0036 (19)
C13	0.104 (4)	0.025 (3)	0.029 (3)	0.014 (3)	0.006 (3)	−0.008 (2)
C14	0.081 (4)	0.030 (3)	0.026 (2)	0.009 (3)	0.001 (2)	−0.001 (2)

### Geometric parameters (Å, °)

**Table:**

Br1—C3	1.888 (4)	C3—C4	1.371 (6)
Br2—C5	1.897 (4)	C4—C5	1.382 (6)
O1—C2	1.348 (5)	C4—H4	0.9300
O1—H1	0.8200	C5—C6	1.374 (6)
O2—C8	1.225 (5)	C6—H6	0.9300
O3—C12	1.355 (5)	C7—H7	0.9300
O3—H3	0.8200	C8—C9	1.486 (5)
O4—H4A	0.847 (10)	C9—C14	1.379 (5)
O4—H4B	0.851 (10)	C9—C10	1.379 (6)
N1—C7	1.275 (5)	C10—C11	1.380 (6)
N1—N2	1.371 (4)	C10—H10	0.9300
N2—C8	1.359 (5)	C11—C12	1.365 (6)
N2—H2	0.898 (10)	C11—H11	0.9300
C1—C6	1.387 (5)	C12—C13	1.375 (6)
C1—C2	1.411 (6)	C13—C14	1.368 (6)
C1—C7	1.446 (6)	C13—H13	0.9300
C2—C3	1.395 (6)	C14—H14	0.9300
			
C2—O1—H1	109.5	N1—C7—C1	120.2 (4)
C12—O3—H3	109.5	N1—C7—H7	119.9
H4A—O4—H4B	108 (2)	C1—C7—H7	119.9
C7—N1—N2	119.5 (4)	O2—C8—N2	120.0 (4)
C8—N2—N1	118.2 (3)	O2—C8—C9	123.9 (4)
C8—N2—H2	124 (4)	N2—C8—C9	116.1 (4)
N1—N2—H2	117 (4)	C14—C9—C10	117.3 (4)
C6—C1—C2	119.4 (4)	C14—C9—C8	124.3 (4)
C6—C1—C7	119.6 (4)	C10—C9—C8	118.4 (4)
C2—C1—C7	120.9 (4)	C9—C10—C11	122.5 (4)
O1—C2—C3	119.1 (4)	C9—C10—H10	118.7
O1—C2—C1	122.8 (4)	C11—C10—H10	118.7
C3—C2—C1	118.1 (4)	C12—C11—C10	119.0 (4)
C4—C3—C2	122.3 (4)	C12—C11—H11	120.5
C4—C3—Br1	119.5 (3)	C10—C11—H11	120.5
C2—C3—Br1	118.2 (3)	O3—C12—C11	122.6 (4)
C3—C4—C5	118.6 (4)	O3—C12—C13	118.1 (4)
C3—C4—H4	120.7	C11—C12—C13	119.3 (4)
C5—C4—H4	120.7	C14—C13—C12	121.3 (4)
C6—C5—C4	121.1 (4)	C14—C13—H13	119.4
C6—C5—Br2	120.0 (4)	C12—C13—H13	119.4
C4—C5—Br2	118.9 (3)	C13—C14—C9	120.6 (4)
C5—C6—C1	120.5 (4)	C13—C14—H14	119.7
C5—C6—H6	119.7	C9—C14—H14	119.7
C1—C6—H6	119.7		

### Hydrogen-bond geometry (Å, °)

**Table:**

*D*—H···*A*	*D*—H	H···*A*	*D*···*A*	*D*—H···*A*
O1—H1···N1	0.82	1.86	2.578 (4)	146.
O3—H3···O2^i^	0.82	1.83	2.642 (4)	173.
O4—H4A···O3^ii^	0.85 (1)	2.04 (1)	2.878 (4)	171 (5)
O4—H4B···O1^i^	0.85 (1)	2.24 (3)	2.969 (5)	144 (4)
N2—H2···O4^iii^	0.90 (1)	2.01 (2)	2.874 (5)	162 (5)

Symmetry codes: (i) −*x*+1, *y*−1/2, −*z*+1/2; (ii) −*x*+1, *y*+1/2, −*z*+1/2; (iii) *x*, −*y*+1/2, *z*−1/2.
